# Correction: Ferdowsi et al. Capsaicin and Zinc Signalling Pathways as Promising Targets for Managing Insulin Resistance and Type 2 Diabetes. *Molecules* 2023, *28*, 2861

**DOI:** 10.3390/molecules30071446

**Published:** 2025-03-25

**Authors:** Parisa Vahidi Ferdowsi, Kiran D. K. Ahuja, Jeffrey M. Beckett, Stephen Myers

**Affiliations:** 1 School of Health Sciences, College of Health and Medicine, University of Tasmania, Newnham Drive, Launceston, TAS 7248, Australia; 2 Children’s Cancer Institute, Lowy Cancer Research Centre, C25/9 High St, Kensington, NSW 2750, Australia

Following publication, concerns were raised regarding the peer-review process related to the publication of this article [1]. Adhering to our standard procedure, the Editorial Board conducted an investigation which determined that, while the peer-review process does comply with MDPI’s Editorial process policy (https://www.mdpi.com/editorial_process), the contribution of one of the reviewers does not comply with MDPI’s Guideline for Reviewers (https://www.mdpi.com/reviewers#_bookmark11) and the expectations of the Editorial Board. As a result, the Editorial Board has decided to remove the contribution of one of the reviewers from the open peer-review record (https://www.mdpi.com/1420-3049/28/6/2861/review_report) and conduct a post-publication peer-review to add a new review report.

Based on the new review report, the authors have made the following revisions to this article [1]:

**Text correction**

The 1st sentence in Section 1, Paragraph 3 “IR is identified as an impaired response of the target tissues, primarily the liver, adipose tissue, and skeletal muscle, to insulin-stimulated glucose uptake.” has been modified to “IR is identified as an impaired response of the target tissues, primarily adipose tissue and skeletal muscle to insulin-stimulated glucose uptake.”.

The 4th sentence in Section 1, Paragraph 9 “Src homology region 2 (SHP2), and extracellular signal-regulated kinase 1/2 (ERK1/2), independent of insulin.” has been modified to “Src homology region 2 (SHP2; also known as SH2 domain-containing protein tyrosine phosphatase 2), and extracellular signal-regulated kinase 1/2 (ERK1/2), independent of insulin.”.

The 10th sentence in Section 2, Paragraph 1 “the AKT substrate of 160 kDa (AS160) has been established as an important stimulator of GLUT4 translocation.” has been modified to “the AKT substrate of 160 kDa (AS160) has been established as an important stimulator of GLUT4 retention.”.

The Figure 1, Figure 2 and Figure 3 ’s captions have been updated and appear below.

**Figure 1.** Insulin signal transduction. Insulin binding to its receptor’s extracellular α subunit causes a conformational change in the β subunit of the receptor, which eventually triggers phosphorylation (designated as P) of intracellular proteins, IRSs, which further activates insulin-mediated downstream events including phosphorylation of signalling proteins PI3K, PIP3, AKT, and AS160, leading to GLUT4 movement and glucose disposal.

**Figure 2.** Pathophysiology of T2DM. Several factors can lead to the development of Type 2 Diabetes Mellitus. These include environmental factors such as lifestyle (physical inactivity, diet, etc), and genetics. These factors can contribute to insulin resistance in muscle, adipose, and liver. Insulin resistance is the precursor to beta cell dysfunction, impaired glucose tolerance, and the development of Type 2 Diabetes Mellitus.

**Figure 3.** CAMK pathway and the downstream signalling events following TRPV1 activation by capsaicin. Activation of the TRPV1 channel by capsaicin triggers an elevation in calcium flux to the cytosol, which initiates calcium signal transduction in the skeletal muscle cell. Calcium binding to CAM induces a structural modification in CAM and forms a calcium/calmodulin complex. This complex controls the activity of CAMKKs and AMPK, which accordingly stimulate signalling events leading to glucose uptake, including CREB and TORCs activation, and the translocation of Glut4 receptors.

The updated Figure 4 appears below.

**Figure 4:**
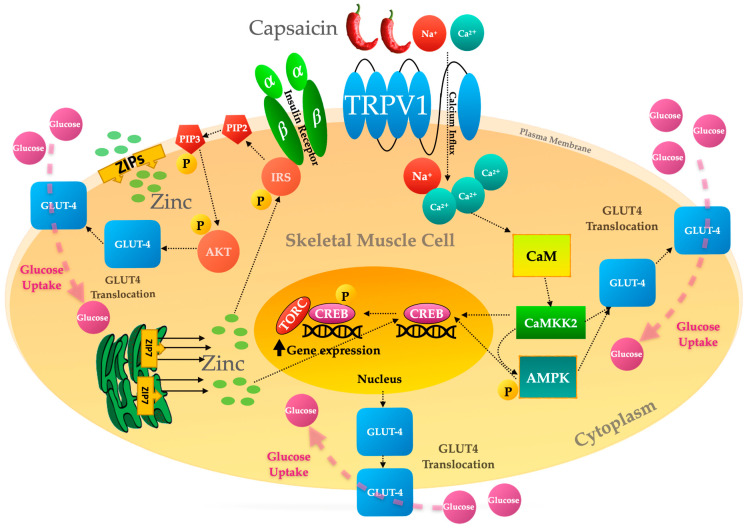
Capsaicin and zinc-induced activation signalling events lead to GLUT4 translocation and glucose uptake in skeletal muscle.

The 4th sentence in Section “*Capsaicin-Induced Signalling Pathways in Glucose Metabolism*”, Section 3, Paragraph 1 “This causes a conformational change in the channel and the opening of a protein pore, permitting calcium release from the extracellular space into the cytoplasm, which can regulate many signalling pathways in the cells.” has been modified to “This causes a conformational change in the channel and the opening of a protein pore, permitting calcium from the extracellular space to enter the cytoplasm, which can regulate many signalling pathways in the cells.”.

In Section 3, “CAMKKs” has been modified to “CAMKs”,”CAMKK2” has been modified to “CAMK2”.

The 3rd sentence in Section “*Zinc-Induced Signalling Pathways in Glucose Metabolism*”, Section 4, Paragraph 2 “This activation leads to the breakdown of PIP2, which generates two important second messengers, inositol 1,4,5-trisphosphate (IP3) and diacylglycerol (DAG), which work together to promote calcium release from intracellular stores.” has been modified to “This activation leads to the breakdown of PIP2, which generates two important second messengers, inositol 1,4,5-trisphosphate (IP3) and diacylglycerol (DAG), to promote calcium release from intracellular stores.”.

The authors state that the scientific conclusions are unaffected. This correction was approved by the Academic Editor. The original publication has also been updated.
